# Risks of Recurrent Cardiovascular Events and Mortality in 1-Year Survivors of Acute Myocardial Infarction Implanted with Newer-Generation Drug-Eluting Stents

**DOI:** 10.3390/jcm10163642

**Published:** 2021-08-17

**Authors:** Sungmin Lim, Eun Ho Choo, Ik Jun Choi, Kwan Yong Lee, Su Nam Lee, Byung-Hee Hwang, Chan Joon Kim, Mahn-Won Park, Jong-Min Lee, Chul Soo Park, Hee-Yeol Kim, Ki-Dong Yoo, Doo Soo Jeon, Ho Joong Youn, Wook Sung Chung, Min Chul Kim, Myung Ho Jeong, Hyeon Woo Yim, Youngkeun Ahn, Kiyuk Chang

**Affiliations:** 1 Division of Cardiology, Department of Internal Medicine, Uijeongbu St. Mary’s Hospital, College of Medicine, The Catholic University of Korea, Seoul 06591, Korea; mdsungminlim@gmail.com (S.L.); godandsci@catholic.ac.kr (C.J.K.); leejongm@catholic.ac.kr (J.-M.L.); 2 Division of Cardiology, Department of Internal Medicine, Seoul St. Mary’s Hospital, College of Medicine, The Catholic University of Korea, Seoul 06591, Korea; cmcchu@catholic.ac.kr (E.H.C.); cycle0210@naver.com (K.Y.L.); hbhmac@catholic.ac.kr (B.-H.H.); younhj@catholic.ac.kr (H.J.Y.); chungws@catholic.ac.kr (W.S.C.); 3 Division of Cardiology, Department of Internal Medicine, Incheon St. Mary’s Hospital, College of Medicine, The Catholic University of Korea, Seoul 06591, Korea; mrfasthand@catholic.ac.kr (I.J.C.); coronary@catholic.ac.kr (D.S.J.); 4 Division of Cardiology, Department of Internal Medicine, St Vincent’s Hospital, College of Medicine, The Catholic University of Korea, Seoul 06591, Korea; yellow-night@hanmail.net (S.N.L.); yookd@catholic.ac.kr (K.-D.Y.); 5 Division of Cardiology, Department of Internal Medicine, Daejeon St. Mary’s Hospital, College of Medicine, The Catholic University of Korea, Seoul 06591, Korea; pmw6193@catholic.ac.kr; 6 Division of Cardiology, Department of Internal Medicine, Yeouido St. Mary’s Hospital, College of Medicine, The Catholic University of Korea, Seoul 06591, Korea; charlie@catholic.ac.kr; 7 Division of Cardiology, Department of Internal Medicine, Bucheon St. Mary’s Hospital, College of Medicine, The Catholic University of Korea, Seoul 06591, Korea; cumckhy@catholic.ac.kr; 8 Division of Cardiology, Department of Internal Medicine, Chonnam National University Hospital, Chonnam National University School of Medicine, Gwangju 61469, Korea; kmc3242@hanmail.net (M.C.K.); myungho@chollian.net (M.H.J.); 9 Department of Preventive Medicine, Clinical Research Coordinating Center, College of Medicine, The Catholic University of Korea, Seoul 06591, Korea; y1693@catholic.ac.kr; 10 Catholic Research Institute for Intractable Cardiovascular Disease (CRID), College of Medicine, The Catholic University of Korea, Seoul 06591, Korea

**Keywords:** acute myocardial infarction, drug-eluting stents, mortality, percutaneous coronary intervention, prognosis

## Abstract

Current treatments for acute myocardial infarction (AMI) have dramatically improved clinical outcomes during the first year after AMI. Less is known, however, about the subsequent risks of recurrent cardiovascular events and mortality in patients who survive 1 year after AMI. The purpose of the present study is to evaluate long-term clinical outcomes in 1-year AMI survivors who were implanted with newer-generation drug-eluting stents (DESs) since 2010. The COREA-AMI (CardiOvascular Risk and idEntificAtion of potential high-risk population in AMI) registry consecutively enrolled AMI patients who underwent percutaneous coronary intervention (PCI), and patients who received newer-generation DESs since 2010 were analyzed. The primary endpoint was major adverse cardiovascular events (MACEs), and secondary endpoint was all-cause mortality. Of 6242 AMI patients, 5397 were alive 1 year after the index procedure. The cumulative incidence of MACEs and all-cause death 1 to 7 years after AMI were 28.4% (annually 4–6%) and 20.2% (annually 3–4%), respectively. Multivariate analysis showed that uncontrolled systolic blood pressure (SBP) and serum low-density lipoprotein cholesterol (LDL-C) concentration, as well as traditional risk factors, were associated with MACEs and all-cause death. Recurrent non-fatal myocardial infarction, ischemic stroke, and bleeding events within 1 year were significantly associated with all-cause death. The risks of adverse cardiovascular events and death remain high in AMI patients more than 1 year after the index PCI with newer-generation DESs. Traditional risk factors, uncontrolled SBP and LDL-C, and non-fatal adverse events within 1 year after the index procedure strongly influence long-term clinical outcomes.

## 1. Introduction

Acute myocardial infarction (AMI) is a common and fatal manifestation of ischemic heart disease. Although deaths from cardiovascular disease are increasing as a result of population growth and aging of the population [1,2], the age-standardized death rate from AMI has been declining in most developed countries [3]. These improvements have been attributed to many potential reasons, including the early diagnosis and treatment of AMI, the widespread use of cardiac catheterization and revascularization, the appropriate use of antithrombotics, secondary preventive therapies, development of the coronary care unit, and advances in critical care, as well as a decline in the prevalence of smoking and modifications in physical activities [3,4].

Despite these advances in diagnosis and management, AMI remains a major public health concern worldwide. Although current treatment guidelines have been updated and effective therapies recommended [5,6,7], many recommendations have focused on patient management during hospitalization or the first year after diagnosis, as most clinical trials have assessed the efficacy of beneficial strategies during the acute phase or the mid-term period, usually within 1 or 2 years [8,9,10]. However, the improved rates of short-term survival after AMI have led to evaluations of the rates of and risk factors for adverse events occurring over longer periods of time. Although several studies have demonstrated improved long-term prognosis in short-term survivors, most of these trials were conducted in the 2000s and included patients who did not undergo revascularization or received older-generation stents [11,12,13]. At present, most AMI patients are implanted with newer-generation drug-eluting stents (DESs) and take guideline-directed medical therapy [14,15]. Therefore, the purpose of the present study was to evaluate long-term clinical outcomes, for up to 7 years, in 1-year AMI survivors who were implanted with newer-generation DESs since 2010.

## 2. Materials and Methods

### 2.1. Study Population

The COREA-AMI (CardiOvascular Risk and idEntificAtion of potential high-risk population in Acute Myocardial Infarction) registry was designed to evaluate the real-world, long-term clinical outcomes of all consecutive patients with AMI who were evaluated at nine major university cardiac centers located throughout South Korea. These nine hospitals have shared, web-based registries enrolling AMI patients. The COREA-AMI I registry included AMI patients who underwent percutaneous coronary intervention (PCI) from January 2004 to December 2009. The COREA-AMI II registry extended the follow-up period and included additional patients from January 2010 to August 2014. Factors recorded in this large observational registry include demographic, clinical, and procedural data, and short-term and long-term clinical outcomes as long as possible by 2019. All of the included patients were over 20 years old and were treated with PCI. Patients managed with a conservative strategy were excluded. The study protocol conformed with the Declaration of Helsinki regarding investigations in humans and was approved by the Institutional Review Board of each participating center. This registry has been registered on www.ClinicalTrials.gov as NCT02806102 (accessed on 20 June 2016).

Patients were diagnosed with AMI if their concentrations of cardiac biomarkers were at least one unit above the 99th percentile with temporal variations and at least one of the following indications: symptoms of ischemia, new or apparently new significant ST-segment-T wave changes or new left bundle branch block, electrocardiographic indications of pathological Q waves, imaging evidence of new loss of viable myocardium or new regional wall motion abnormality, or an angiographically detected intracoronary thrombus [16]. Patients were divided into two groups based on electrocardiography findings: those with ST-segment elevation myocardial infarction (STEMI) and non-ST-segment elevation myocardial infarction (NSTEMI). Patients assigned to the STEMI group including those with typical ST-segment elevation in two contiguous leads and atypical electrocardiographic presentations, such as bundle branch block, isolated posterior wall myocardial infarction (MI), or ST-segment elevation in lead aVR, with appropriate clinical and angiographic findings; all other patients were assigned to the NSTEMI group.

**Figure 1:**
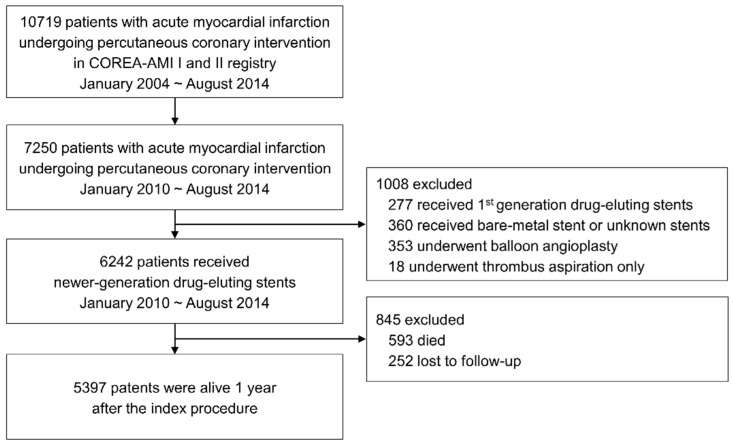
Study flow chart.

Figure 1 depicts the study flow. Between January 2004 and August 2014, 10,719 patients were diagnosed with AMI and underwent PCI. Until 2009, most were implanted with first-generation DESs. The present study evaluated patients who received newer-generation DESs from January 2010 to August 2014. Patients implanted with a bare-metal stent or first-generation DES, even if also implanted with a newer-generation DES, were excluded. Of the 6242 AMI patients who were implanted a with newer-generation DES, 593 died, and 252 were lost to follow-up within 1 year after the index procedure. Therefore, this study analyzed 5397 patients who survived for 1 year after implantation of newer-generation DESs.

### 2.2. Treatment and Data Collection

Coronary angiography and interventions were performed according to standard guidelines [17,18,19,20]. Patients naïve to aspirin and P2Y12 inhibitor were administered loading doses of 250–500 mg aspirin and 300–600 mg clopidogrel, 180 mg ticagrelor, or 60 mg prasugrel. The revascularization strategy, techniques, selection of devices, and adjunctive antithrombotic therapy were at the discretion of the operators. Treatments recommended after PCI included maintenance doses of aspirin (usually 100 mg once daily) and P2Y12 inhibitors (clopidogrel 75 mg once daily, ticagrelor 90 mg twice daily, or prasugrel 10 mg once daily), with the intensity and duration of dual antiplatelet therapy at the discretion of the physician. Optimal pharmacological therapy, including statins, beta-blockers, or renin-angiotensin system inhibitors, was recommended after the intervention, according to standard guidelines. Doses were titrated and medications changed at the index admission and during follow-up according to each patient’s condition.

All data were collected on a web-based system after eliminating personal information. Independent research personnel collected baseline clinical, laboratory, and medication data, and independent trained reviewers and interventional cardiologists assessed angiographic and procedural data. Vital signs were obtained from chart reviews at hospital admission and discharge and at each visit to the outpatient clinic. Follow-up laboratory data were determined at the discretion of each physician. Blood pressure and lipid profiles were obtained by reviewing patients’ charts. Clinical events and outcomes were obtained from electrical medical records and telephone conversations. All the adverse clinical events of interest were determined by source documents and were confirmed centrally by the committee of the Cardiovascular Center of Seoul St. Mary’s Hospital, Seoul, Republic of Korea. Mortality was verified by determining whether the patient had been removed from the National Health Insurance Service, the single government-managed insurance program in Korea that includes almost the entire population of the country. The final dataset was managed by independent statisticians of the Clinical Research Coordinating Center and sealed with a code by a Clinical Research Associate.

### 2.3. Endpoints and Definitions

Clinical outcomes from 1 to 7 years after the index PCI were analyzed in 1-year survivors of AMI who underwent newer-generation DES implantation. The primary endpoint of this study was the incidence of major adverse cardiovascular events (MACEs), defined as cardiovascular death, non-fatal MI, non-fatal ischemic stroke, and any revascularization. The secondary endpoints were all-cause death; individual components of MACEs, including cardiovascular death, non-fatal MI, non-fatal ischemic stroke, and revascularization; and bleeding events, defined as Bleeding Academic Research Consortium (BARC) 2, 3, and 5 [21].

Cardiovascular death was defined as death resulting from AMI, sudden cardiac death, heart failure, stroke, or other vascular cause. Deaths without definite non-cardiovascular causes were considered cardiovascular deaths. Recurrent MI was defined as elevated cardiac marker(s) with evidence of ischemia, such as a typical symptom, electrocardiographic changes, or appropriate anatomical or functional results. Stroke was defined as an episode of focal or global neurologic dysfunction related to the brain, spinal cord, or retinal vascular injury as a result of infarction or hemorrhage. Any revascularization was defined as any unscheduled repeat PCI to any coronary lesions or unplanned coronary artery bypass surgery. Uncontrolled systolic blood pressure (SBP) was defined as mean office SBP ≥ 130 mmHg within 1 year after index PCI during follow-up, whereas uncontrolled low-density lipoprotein cholesterol (LDL-C) was defined as LDL-C ≥ 100 mg/dL at 1 year after index PCI.

### 2.4. Statistical Analysis

Continuous variables were reported as the mean ± standard deviation (SD) or median (interquartile range (IQR)) and analyzed by independent sample *t*-tests or Mann-Whitney *U*-tests, according to their distribution. Categorical variables were reported as a number (percent or rate) and were analyzed by the chi-square test or Fisher’s exact test, as appropriate. The cumulative yearly incidence of primary and secondary endpoints was evaluated by the Kaplan-Meier method and compared by log-rank tests. Landmark analyses for events that occurred within the time points were performed. A Cox proportional hazard model was applied to analyze hazard ratios (HRs) and 95% confidence interval (CI) of covariates for endpoints. Univariate analyses included all variables in the table of baseline characteristics. To adjust for confounding factors, multivariate analyses were performed using variables significantly (*p* < 0.1) associated with clinical events in univariate analyses. Age cutoffs were at 55 and 65 years, based on approximate quartiles. The clinical impact of non-fatal events (MI, ischemic stroke, any revascularization, and bleeding) within 1 year after the index procedure on all-cause death beyond 1 year was analyzed by adjusting for all covariates with *p* < 0.1 on univariate analysis. All analyses were two-tailed, with clinical significance defined as *p* < 0.05. All statistical analyses were performed using SAS version 9.8 (SAS Institute Inc., Cary, NC, USA), SPSS version 20.0 (SPSS Inc., Chicago, IL, USA), and R version 3.6.1 (R Foundation for Statistical Computing, Vienna, Austria) statistical software.

## 3. Results

### 3.1. Baseline Characteristics

Of the 7250 patients with AMI who underwent PCI between January 2010 and August 2014, 6242 (86.1%) received newer-generation DESs; of the latter, 845 patients died or were lost to follow-up within 1 year after the index procedure, and 5397 survived. Everolimus-eluting stents were implanted in 2771 (51.3%), zotarolimus-eluting stents were implanted in 1189 (22.0%), and biolimus-eluting stents were implanted in 1053 (19.5%) patients. The other 384 (7.1%) patients received heterogeneous stent types. The baseline characteristics of patients who underwent the index procedure and those who survived for 1-year are summarized in Table 1. The mean age of the survivors was 62.8 ± 12.4 years, and 73.7% were men. Mean SBP and diastolic blood pressure (DBP) were 113.3 ± 14.7 and 69.7 ± 9.8 mmHg, respectively, at discharge of index hospitalization, and 116.4 ± 13.4 and 68.0 ± 9.1 mmHg, respectively, within 1 year in 1-year survivors. The concentrations of total cholesterol, triglycerides, high-density lipoprotein cholesterol, and LDL-C were 177.5 ± 44.6, 124.5 ± 99.4, 40.5 ± 10.9, and 118.4 ± 39.3 mg/dl, respectively, at index admission, and 146.5 ± 37.2, 141.8 ± 91.5, 42.6 ± 11.3, and 83.6 ± 31.5 mg/dl, respectively, at 1 year in 1-year survivors. More than 98% of patients received dual antiplatelet therapy, 94.2% of patients were prescribed statin, and 86.4% and 75.3% of patients received beta-blocker and renin-angiotensin system inhibitor, respectively (Supplementary Materials Table S1).

### 3.2. Clinical Outcomes beyond 1 Year

**Figure 2:**
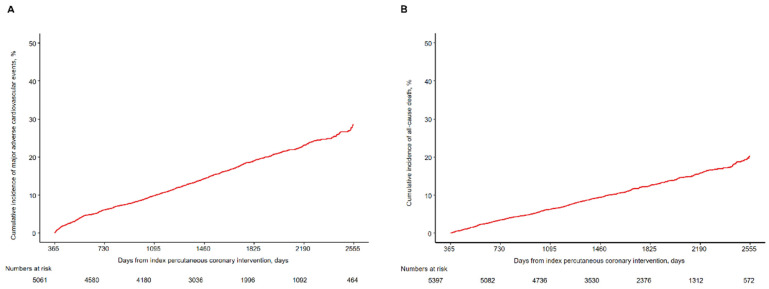
Kaplan-Meier analyses of cumulative incidence of (**A**) major adverse cardiovascular events and (**B**) all-cause death.

The median follow-up duration was 1575 days (interquartile range 1102 to 2097 days). Table 2 shows the numbers of events and Kaplan-Meier estimates of events in the study population. The cumulative incidence of MACEs and all-cause mortality 1 to 7 years after index PCI were 28.5% (928 events/5061 patients) and 20.2% (684 deaths/5397 patients), respectively (Figure 2). The annual risk of MACEs more than 1 year after index PCI was about 4–6% and the annual risk of all-cause mortality after 1 year was about 3–4%. Non-fatal MI, non-fatal stroke, any revascularization, and bleeding events were observed even in patients who did not experience events during the first year after index PCI. Although the annual risk of events during the first year was higher than those in subsequent years, the steady occurrence of events resulted in a higher overall incidence over time.

Clinical outcomes were also analyzed in patients subgrouped by age. The numbers of events and Kaplan-Meier estimates are shown in Supplementary Materials Table S2. The annual risks of MACEs more than 1 year after index PCI in patients aged <55, 55–64, 65–74, and ≥75 years were about 2.2–4.0%, 3.3–6.0%, 4.0–7.0%, and 7.5–11.8%, respectively, whereas the annual risks of all-cause death in these four subgroups were 0.6–0.9%, 1.3–3.8%, 3.5–4.7%, and 7.8–11.9%, respectively. The incidence of MACEs, all-cause and cardiovascular deaths, ischemic stroke, and bleeding increased with increasing age, whereas the incidence of MI and any revascularization did not. Kaplan-Meier analyses of MACEs and all-cause death in patients subgrouped by age showed that the log-rank *p* value for all age categories was <0.001 for both more than 1 year after the index procedure (Supplementary Materials Figure S1).

Predictors of MACEs and all-cause death beyond 1 year.

Multivariate analyses were performed to identify predictors of MACEs and all-cause death more than 1 year after index PCI, as shown by unadjusted (Supplementary Materials Table S3) and adjusted (Table 3) HRs and CIs. Age, hypertension, diabetes mellitus, current smoker, previous PCI, reduced left ventricular ejection fraction (LVEF), anemia, renal insufficiency, multivessel coronary artery disease (CAD), mean stent diameter < 3 mm, uncontrolled SBP, and uncontrolled LDL-C were found to be significantly associated with MACEs after 1 year. By contrast, age, male sex, diabetes mellitus, reduced LVEF, anemia, renal insufficiency, multivessel CAD, uncontrolled SBP, and uncontrolled LDL-C were significantly associated with all-cause death after 1 year.

Multivariate analyses were also performed to identify predictors of MACEs and all-cause death (Supplementary Materials Table S4) more than 1 year after index PCI as a function of patient age. Anemia, renal insufficiency, multivessel CAD, and uncontrolled LDL-C were found to be significantly associated with MACEs in patients aged <65 years, whereas age, Killip classification III~IV, reduced LVEF, multivessel CAD, multivessel intervention, uncontrolled SBP, and uncontrolled LDL-C were associated with MACEs in patients aged ≥65 years. By contrast, lower body mass index, diabetes mellitus, reduced LVEF, anemia, renal insufficiency, and uncontrolled SBP were associated with all-cause death in patients aged <65 years, whereas age, male sex, Killip classification III or IV, reduced LVEF, renal insufficiency, uncontrolled SBP, and uncontrolled LDL-C were significant predictors of all-cause death in patients aged ≥65 years. Kaplan-Meier analysis of all-cause death in men and women according to age found that the cumulative unadjusted risk of all-cause death in patients aged <65 years was significantly lower in men than in women (unadjusted HR 0.63, 95% CI 0.40–0.99, *p* = 0.044), but this difference was not statistically significant after multivariate adjustment (adjusted HR 1.50, 95% CI 0.84–2.69, *p* = 0.172) (Supplementary Materials Figure S2). By contrast, the risk of all-cause death was significantly higher in men than in women aged ≥65 years (unadjusted HR 1.24, 95% CI 1.04–1.47, *p* = 0.014; unadjusted *p* for interaction = 0.006), a difference that remained statistically significant after multivariate adjustment (adjusted HR 1.82, 95% CI 1.38–2.42, *p* < 0.001; adjusted *p* for interaction = 0.114).

Among the 1-year survivors, 80 (1.5%) experienced non-fatal MI, 58 (1.1%) experienced non-fatal ischemic stroke, 259 (4.8%) required revascularization, 34 (0.6%) experienced stent thrombosis, and 312 (5.8%) had BARC 2, 3, and 5 bleeding within 1 year after the index PCI. Non-fatal MI, ischemic stroke, and bleeding within 1 year were significantly associated with all-cause death beyond 1 year, even after adjustment of significant covariates (Table 4), whereas any revascularization (unadjusted HR 1.02, 95% CI 0.73–1.43, *p* = 0.89) and stent thrombosis (unadjusted HR 1.91, 95% CI 0.95–3.83, *p* = 0.07) was not related to all-cause death.

## 4. Discussion

This real-world AMI registry described the long-term clinical outcomes and their predictors in 1-year AMI survivors who underwent PCI with newer-generation DESs and received guideline-directed medical therapy. The annual risk of MACEs and all-cause death more than 1 year after index PCI were 4–6% and 3–4%, respectively, and the cumulative incidence from 1 to 7 years was 28.4% and 20.2%, respectively. Uncontrolled SBP and serum LDL-C concentration, as well as traditional risk factors, were found to be associated with higher risks of MACEs and all-cause death after 1 year. In addition, 1-year survivors who experienced non-fatal MI, ischemic stroke, or bleeding within 1 year were found to be at increased risk of all-cause death beyond 1 year.

Previous registry data and randomized controlled trials have reported long-term cardiovascular risks in patients with AMI. Table 5 lists the long-term clinical outcomes of representative studies and of the present registry [13,22,23,24,25,26,27,28,29,30]. The long-term risks of combined endpoints, cardiovascular death, and all-cause death in our registry were similar to those in some Western registries or in Asian registries [22,26], whereas the risks in our registry were lower than those in several Western national health records that included all MI patients [23,24,25]. Later national health records studies included very older patients and more females, with fewer than two-thirds being treated with PCI or bypass surgery. The cumulative rates of combined endpoints, cardiovascular death, and all-cause death in the present study were higher than those in randomized controlled trials [13,27,28,29,30]. Although the risks of MI and stroke were lower in the present study than in Western national health records studies and one randomized trial that included high-risk patients without history of PCI [13,23,25], the rates in our study were similar to those in Asian registry studies and other randomized trials that included only patients implanted with DES [26,27,28,29,30]. Our real-world registry included all consecutive AMI patients who underwent PCI, with the present study showing real-world clinical outcomes in 1-year survivors of AMI who received newer-generation DESs since 2010. Although direct statistical comparisons could not be performed, our findings demonstrate that, compared with previous various observational and randomized studies, the rates of cardiovascular and all-cause mortality were similar or higher, while the rates of MI and stroke were similar to studies that included only patients implanted with DES. Moreover, our study found that long-term cardiovascular risks persist in AMI patients who survive 1 year after an index PCI, even when implanted with newer-generation DESs.

Multivariate analyses demonstrated two important findings in patients who survived 1 year after AMI: First, in addition to traditional important risk factors, such as age, reduced LVEF, renal insufficiency, and multivessel CAD, two controllable risk factors, uncontrolled SBP and LDL-C, were significantly associated with poorer clinical outcomes and higher rates of all-cause death. Second, non-fatal MI, ischemic stroke, and bleeding events within 1 year from an index PCI were significantly associated with all-cause death more than 1 year after AMI. Control of BP and lipids has been shown to be important in patients with cardiovascular disease [31,32], and early adverse events were found to be associated with poorer long-term clinical outcomes [33,34]. The present findings emphasize the importance of controlling BP and LDL more than 1 year after AMI, even in patients implanted with the newer-generation DESs. Large randomized trials and/or other large-scale registry studies are needed to confirm the importance of BP and LDL-C control in AMI survivors 1 year after index PCI involving the implantation of newer-generation DESs.

Sex differences in long-term mortality after AMI have been reported. For example, unadjusted analyses showed higher mortality rates in women than in men. However, mixed results have been reported after adjustment for confounding factors [35]. Although the present study found that long-term unadjusted risk of MACEs was lower in men than in women who survived 1-year after newer-generation DES implantation, this risk was similar in men and women after multivariate adjustment. By contrast, the long-term unadjusted risk of all-cause death was lower in men than in women, whereas multivariate analysis showed a higher risk in men. These results were apparently related to the increased unadjusted and adjusted HRs in men aged ≥65 years. These patterns are similar to a study showing that the adjusted HR for 20-year mortality was higher in men than in women [36]. In addition, 2018 data from the Korean Statistical Information Service (http://kosis.kr (accessed on 14 October 2020)) on persons aged ≥65 years in the general population found that the crude mortality rate was significantly higher in men than in women (3551.9 per 100,000 vs. 2779.8 per 100,000, risk ratio 1.28, 95% CI 1.22–1.34, *p* < 0.001). These findings were in good agreement with the difference we observed in the long-term incidence of all-cause death between men and women aged ≥65 years. Nevertheless, additional studies are required to assess differences in mortality between male and female AMI patients following PCI with newer-generation DESs.

This study had several limitations. First, this study was a registry-based observational study, with its inherent limitations and inability to adjust for unmeasurable confounders. In addition, the frequencies of missing values in registry studies differ among variables, such that inclusion of these missing values may have affected the study results. Second, despite intensive chart review and telephone interviews, some adverse clinical events may have been underestimated and some patients were lost to follow-up. However, the rate of all-cause death was quite accurate, as almost all deaths could be validated by the removal of patients from the National Health Insurance Service. Third, uncontrolled diabetes mellitus could not be evaluated because more than half were missing the value of Hb A1c. Forth, this study only patients who underwent implantation of newer-generation DESs were included. Thus, these results should be interpreted with caution in patients managed with other treatment options.

## 5. Conclusions

The risks of adverse cardiovascular events and all-cause death persist for several years in AMI patients who survive 1 year after PCI with newer-generation DESs. Several traditional risk factors were found to be predictors of adverse events. In addition, uncontrolled risk factors and non-fatal adverse events within 1 year were also associated with MACEs and mortality more than 1 year after index PCI. Physicians should be concerned with assessing and controlling risk factors and preventing non-fatal adverse events in patients with AMI.

## Figures and Tables

**Table 1:** Baseline characteristics of the study population at the time of and 1 year after the index percutaneous coronary intervention procedure.

Variables	At Index Procedure (*n* = 6242)	At 1 Year (*n* = 5397)
Age, years	63.78 ± 12.63	62.84 ± 12.39
<55	1640 (26.3)	1530 (28.3)
55–64	1514 (24.3)	1367 (25.3)
65–74	1688 (27.0)	1448 (26.8)
≥75	1400 (22.4)	1052 (19.5)
Men	4508 (72.2)	3975 (73.7)
Body mass index, kg/m^2^	24.11 ± 3.27	24.22 ± 3.21
Body mass index ≥ 25	2236 (36.8)	2013 (37.9)
Hypertension	3291 (52.7)	2770 (51.3)
Diabetes mellitus	1985 (31.8)	1641 (30.4)
Dyslipidemia	1196 (19.2)	1082 (20.0)
Current smoker	2477 (39.7)	2211 (41.0)
Family history of coronary artery disease	184 (2.9)	163 (3.0)
Previous myocardial infarction	213 (3.4)	169 (3.1)
Previous percutaneous coronary intervention	402 (6.4)	325 (6.0)
Previous coronary artery bypass graft	25 (0.4)	18 (0.3)
Previous cerebrovascular accident	470 (7.5)	368 (6.8)
Atrial fibrillation	283 (4.5)	210 (3.9)
Clinical presentation		
STEMI	3243 (52.0)	2756 (51.1)
NSTEMI	2999 (48.0)	2641 (48.9)
Killip classification III–IV	965 (15.8)	663 (12.5)
Left ventricular ejection fraction, %	61.12	62.89
Left ventricular ejection fraction < 40	695 (11.7)	510 (9.7)
Hemoglobin, g/dL	13.66 ± 2.14	13.81 ± 2.05
Anemia *	1788 (28.7)	1388 (25.7)
Estimated glomerular filtration rate, mL/min/m^2^	76.58 ± 25.96	79.14 ± 24.61
Renal insufficiency ^†^	1568 (25.2)	1149 (21.3)
Number of coronary arteries involved		
One	2806 (45.0)	2489 (46.1)
Two	2108 (33.8)	1817 (33.7)
Three	1328 (21.3)	1091 (20.2)
Multivessel coronary artery disease	3436 (55.0)	2908 (53.9)
Culprit coronary artery		
Left anterior descending	2999 (48.0)	2578 (47.8)
Left circumflex	1056 (16.9)	952 (17.6)
Right coronary	1951 (31.3)	1695 (31.4)
Left main	233 (3.7)	169 (3.1)
Left anterior descending artery or left main culprit vessel	3232 (51.8)	2747 (50.9)
Number of coronary arteries treated ≥2	1841 (29.5)	1597 (29.6)
Drug-eluting stents		
Everolimus-eluting stents	3262 (52.3)	2771 (51.3)
Zotarolimus-eluting stents	1364 (21.9)	1189 (22.0)
Biolimus-eluting stents	1175 (18.8)	1053 (19.5)
Mixed stents	441 (7.1)	384 (7.1)
Total number of stents	1.58 ± 0.83	1.59 ± 0.84
≥3 stents	851 (13.6)	745 (13.8)
Mean stent diameter, mm	2.75 ± 0.85	2.71 ± 0.85
<3 mm	1843 (29.5)	1543 (28.6)
Total stent length, mm	33.55 ± 20.06	33.71 ± 20.30
>60 mm	629 (10.1)	554 (10.3)
Uncontrolled systolic blood pressure within 1 year	812 (14.5)	705 (13.4)
Uncontrolled LDL-cholesterol within 1 year	1066 (25.4)	1039 (25.3)

Data are shown as mean ± standard deviation or number (percent). Abbreviations: LDL, low-density lipoprotein; NSTEMI, non-ST-segment elevation myocardial infarction; STEMI, ST-segment elevation myocardial infarction. * Hemoglobin < 12 g/dL in women and <13 d/dL in men. ^†^ Estimated glomerular filtration rate using Chronic Kidney Disease Epidemiology Collaboration equation < 60 mL/min/m^2^.

**Table 2:** Clinical outcomes according to time periods after percutaneous coronary intervention.

Outcomes	0–1 Year	1–7 Years	1–2 Years	2–3 Years	3–4 Years	4–5 Years	5–6 Years	6–7 Years
Major adverse cardiovascular event *	862/6242 (14.1 (13.2–15.0))	928/5061 (28.5 (26.4–30.5))	301/5061 (6.1 (5.4–6.8))	177/4580 (3.9 (3.4–4.5))	181/4180 (4.9 (4.2–5.6))	140/3036 (5.5 (4.6–6.4))	78/1996 (5.0 (3.9–6.2))	51/1092 (7.1 (5.1–9.0))
All-cause death	593/6242 (9.6 (8.9–10.4))	684/5397 (20.2 (18.5–21.9))	181/5397 (3.4 (2.9–3.9))	146/5082 (2.9 (2.4–3.4))	140/4736 (3.3 (2.8–3.9))	101/3530 (3.4 (2.7–4.0))	70/2376 (3.8 (2.9–4.7))	46/1312 (5.3 (3.8–6.8))
Cardiovascular death	507/6242 (8.3 (7.6–8.9))	494/5397 (15.4 (13.8–17.0))	123/5397 (2.3 (1.9–2.7))	107/5082 (2.1 (1.7–2.5))	106/4736 (2.6 (2.1–3.0))	69/3530 (2.3 (1.8–2.9))	53/2376 (2.9 (2.1–3.7))	36/1312 (4.2 (2.8–5.6))
Non-fatal myocardial infarction	92/6242 (1.6 (1.3–1.9))	130/5317 (4.1 (3.2–4.9))	40/5317 (0.8 (0.5–1.0))	21/4971 (0.4 (0.2–0.6))	21/4614 (0.5 (0.3–0.7))	28/3420 (0.9 (0.6–1.3))	16/2295 (0.9 (0.5–1.4))	4/1264 (0.5 (0.0–1.0))
Non-fatal ischemic stroke	66/6242 (1.1 (0.9–1.4))	80/5339 (2.4 (1.8–3.0))	20/5339 (0.4 (0.2–0.6))	13/5013 (0.3 (0.1–0.4))	21/4660 (0.5 (0.3–0.7))	15/3465 (0.5 (0.3–0.8))	8/2324 (0.5 (0.1–0.8))	3/1277 (0.3 (0.0–0.6))
Any revascularization	280/6242 (5.0 (4.4–5.5))	433/5140 (12.9 (11.4–14.3))	179/5140 (3.6 (3.1–4.1))	70/4673 (1.5 (1.2–1.9))	70/4280 (1.9 (1.4–2.3))	72/3118 (2.8 (2.1–3.4))	25/2051 (1.5 (0.9–2.1))	17/1127 (2.3 (1.2–3.4))
Stent thrombosis ^†^	58/6242 (1.0 (0.7–1.2))	28/5363 (0.2 (0.1–0.2))	6/5363 (0.1 (0.0–0.2))	5/5047 (0.1 (0.0–0.2))	6/4700 (0.2 (0.0–0.3))	10/3498 (0.3 (0.1–0.5))	1/2353 (0.1 (0.0–0.2))	1/1299 (0.1 (0.0–0.3))
BARC 2, 3, and 5 bleeding	406/6242 (6.9 (6.2–7.5))	225/5085 (6.6 (5.6–7.6))	86/5085 (1.7 (1.4–2.1))	43/4726 (1.0 (0.7–1.2))	42/4384 (1.0 (0.7–1.3))	29/3284 (1.1 (0.7–1.5))	18/2223 (1.1 (0.6–1.6))	7/1231 (0.9 (0.2–1.5))

Data are shown as event number/number at risk (Kaplan-Meier estimates (95% confidence interval)). Abbreviation: BARC, Bleeding Academic Research Consortium. * A composite of cardiovascular death, non-fatal myocardial infarction, non-fatal ischemic stroke, and any revascularization. ^†^ Definite or probable stent thrombosis.

**Table 3:** Multivariable Cox regression analysis of factors associated with major adverse cardiovascular events and all-cause death more than 1 year after percutaneous coronary intervention.

Variables	Major Adverse Cardiovascular Event		Variables	ALL-Cause Death	
	Adjusted HR * (95% CI)	*p* Value		Adjusted HR * (95% CI)	*p* Value
Age, years			Age, years		
<55	1		<55	1	
55–64	1.32 (1.03–1.69)	0.028	55–64	2.08 (1.21–3.59)	0.008
65–74	1.39 (1.07–1.80)	0.015	65–74	5.03 (3.02–8.39)	<0.001
≥75	2.02 (1.50–2.72)	<0.001	≥75	9.12 (5.34–15.6)	<0.001
Hypertension	1.20 (1.00–1.44)	0.046	Male sex	1.71 (1.30–2.24)	<0.001
Diabetes mellitus	1.24 (1.04–1.48)	0.016	Diabetes mellitus	1.35 (1.06–1.72)	0.016
Current smoker	1.23 (1.01–1.49)	0.043	Left ventricular ejection fraction < 40%	2.24 (1.68–2.99)	<0.001
Previous percutaneous coronary intervention	1.44 (1.01–2.04)	0.043	Anemia	1.39 (1.08–1.78)	0.011
Left ventricular ejection fraction < 40%	1.41 (1.10–1.81)	0.006	Renal insufficiency	2.07 (1.60–2.68)	<0.001
Anemia	1.23 (1.02–1.48)	0.030	Multivessel coronary artery disease	1.29 (1.00–1.66)	0.048
Renal insufficiency	1.44 (1.18–1.76)	<0.001	Uncontrolled systolic blood pressure	2.17 (1.65–2.86)	<0.001
Multivessel coronary artery disease	1.42 (1.19–1.68)	<0.001	Uncontrolled LDL-cholesterol	1.36 (1.06–1.74)	0.014
Mean stent diameter < 3 mm	1.26 (1.05–1.50)	0.011			
Uncontrolled systolic blood pressure	1.42 (1.12–1.78)	0.003			
Uncontrolled LDL-cholesterol	1.38 (1.17–1.65)	<0.001			

Abbreviations: CI, confidence interval; HR, hazard ratio; LDL, low-density lipoprotein; * Adjusted by all covariates in Table 1 with *p* values < 0.1 on univariate analysis.

**Table 4:** Association between non-fatal events within 1 year and all-cause death more than 1 year after percutaneous coronary intervention.

Non-Fatal Events	Events	Unadjusted HR (95% CI)	*p* Value	Adjusted HR * (95% CI)	*p* Value
Myocardial infarction	80 (1.5)	2.23 (1.44–3.44)	<0.001	1.64 (1.02–2.65)	0.042
Ischemic stroke	58 (1.1)	2.28 (1.37–3.81)	0.002	1.81 (1.01–3.23)	0.046
BARC 2, 3, and 5 bleeding	312 (5.8)	2.44 (1.92–3.09)	<0.001	1.56 (1.19–2.04)	0.001

Data for events are shown as event number (Kaplan-Meier estimates). Abbreviations: BARC, Bleeding Academic Research Consortium; CI, confidence interval; HR, hazard ratio. * Adjusted by all covariates in Table 4 with *p* values < 0.1 on univariate analysis.

**Table 5:** Long-term clinical outcomes in representative registry studies and randomized controlled trials of patients with acute myocardial infarction.

Study	Year of Publication	Study Design with Trial Name	Patients	Number of Patients	Year of Enrollment	Age (Mean Years)	Men (%)	Period of Events	Clinical Outcomes	Clinical Outcomes of the Present Registry during the Same Period
N Danchin et al. [22]	2020	FAST-MI 2005 and 2010	STEMI with pPCI	timely pPCI 1288 late pPCI 830	2005 and 2010	61	78.1	0–5 years	CE (AD, MI, or stroke) 15.7% and 23.5%, AD 11.8% and 20.5% in timely pPCI and in late pPCI, respectively	CE (AD, MI, or stroke) 25.6%, AD 20.9%
C Özcan et al. [23]	2018	Danish national registry	AMI	43,045	2004–2010	68	65.4	1–5 years	CE (CD, MI, or stroke) 21.7%, CD 9.4%, MI 8.4%, stroke 3.9%.	CE (CD, MI, or stroke) 12.6%, CD 9.0%, MI 3.0%, stroke 2.5%.
E Rapsomaniki et al. [24]	2016	4 national health record data	AMI	114,364	2002–2011	77.5~78.6	50.8~58.5	1–4 years	CE (AD, MI, or stroke) 26.0–36.2%.	CE (AD, MI, or stroke) 11.6%.
T Jernberg et al. [25]	2015	Swedish health record data	AMI	76,687	2006–2011	71.5	63.3	1–4 years	CD 11.0%, MI 9.2%, stroke 3.7%, AD 20.1%.	CD 6.9%, MI 1.9%, stroke 1.8%, AD 9.4%.
K Yamaji et al. [26]	2015	CREDO-Kyoto cohort-2	AMI with DES	820	2005–2007	67.5	73	0–5 years	CD 10.0%, MI 5.2%, AD 18.0%.	CD 16.5%, MI 4.8%, AD 20.9%.
J-Y Hahn et al. [27]	2018	RCT, SMART-DATE	ACS with DES (AMI 69%)	2712	2012–2015	62.0~62.2	74.9~75.9	0–1.5 years	CE (AD, MI, or stroke) 4.2–4.7%, CD 1.4–1.8%, MI 0.8–1.8%, stroke 0.8–0.9%, AD 2.6–2.9%.	CE (AD, MI, or stroke) 13.8%, CD 9.3%, MI 2.3%, stroke 1.4%, AD 11.1%.
M Sabeté et al. [28]	2016	RCT, EXAMINATION	STEMI with DES	751	2008–2010	60.8~61.6	82~84	0–5 years	CE (AD, MI, or revascularization) 21%, CE (CD, target vessel MI, TLR) 12%, CD 6%, MI 5%, revascularization 12%.	CE (AD, MI, or revascularization) 30.7%, CE (CD, target vessel MI, TLR) 20.9%, CD 16.5%, MI 4.8%, revascularization 13.4%.
MP Bonaca et al. [13]	2015	RCT, PEGASUS-TIMI 54	AMI with high risk	21,162	2010–2013	65.2~65.4	75.7~76.4	1–4 (to 3–6) years	CE (CD, MI, or stroke) 7.8–9.0%, CD 2.9–3.4%, MI 4.4–5.3%, stroke 1.5–1.9%, AD 4.7–5.2%.	CE (CD, MI, or stroke) 9.2%, CD 6.9%, MI 1.9%, stroke 1.8%, AD 9.4%.
A de Waha et al. [29]	2015	RCT, ISAR-TEST-4 and LEADERS	STEMI with DES	497	2006–2008	62.5~63.1	72.3~73.5	1–4 years	CE (CD, MI, or TLR) 5.2–8.6%, CD 2.3–3.9%, MI 0.8–2.4%, AD 4.0–6.0%.	CE (CD, MI, or TLR) 9.2%, CD 6.9%, MI 1.9%, AD 9.4%.
L Holmvang et al. [30]	2013	RCT, DEDICATION	STEMI with DES	313	2005–2006	62~63	73~74	8 months–5 years	CE (CD, MI, or TLR) 7.7%, CD 3.5%, MI 3.8%, AD 11.2%.	CE (CD, MI, or TLR) 14.3%, CD 10.7%, MI 3.6%, AD 14.5%.
The present study		COREA-AMI registry	AMI with newer-generation DES	5397	2010–2014	63.8	72.2	1–7 years	CE (CD, MI, stroke, or revascularization) 28.5%, AD 20.2%	

Abbreviations: ACS, acute coronary syndrome; AD, all-cause death; AMI, acute myocardial infarction; CD, cardiac death; CE, combined endpoints; CREDO, Coronary Revascularization Demonstrating Outcome Study; DES, drug-eluting stent; DEDICATION, Drug Eluting and Distal Protection in Acute Myocardial Infarction; EXAMINATION, clinical Evaluation of the Xience-V stent in Acute Myocardial INfArcTION; FAST-MI, The French registry of Acute ST-elevation and non-ST-elevation Myocardial Infarction; ISAR-TEST-4, the Intracoronary Stenting and Angiographic Results: Test Efficacy of 3 Limus-Eluting Stents; LEADERS, Limus Eluted from A Durable versus ERodable Stent coating; MI, myocardial infarction; pPCI, primary percutaneous coronary intervention; RCT, randomized controlled trial; SMART-DATE, the Smart Angioplasty Research Team safety of 6-month duration of Dual Antiplatelet Therapy; STEMI, ST-segment elevation myocardial infarction; TLR, target lesion revascularization.

## Data Availability

Data available on request due to restrictictions.

## Supplementary Materials

The following are available online at https://www.mdpi.com/article/10.3390/jcm10163642/s1, Figure S1: Kaplan-Meier analyses of cumulative incidence of (A) major adverse cardiovascular events and (B) all-cause death according to patient age, Figure S2: Kaplan-Meier analyses of cumulative incidence of all-cause death in men and women 1-year AMI survivors aged <65 and ≥65 years, Table S1: Medications prescribed to patients. Table S2: Clinical outcomes according to patient age and time periods after percutaneous coronary intervention, Table S3: Univariate Cox regression analysis of factors associated with major adverse cardiovascular events and all-cause death more than 1 year after percutaneous coronary intervention, Table S4: Adjusted hazard ratios of factors associated with major adverse cardiovascular events and all-cause death more than 1 year after percutaneous coronary intervention according to patient age.
