# Supplementary material for: Risks of Recurrent Cardiovascular Events and Mortality in 1-Year Survivors of Acute Myocardial Infarction Implanted with Newer-Generation Drug-Eluting Stents
Source: J Clin Med. 2021 Aug 17;10(16):3642. doi: 10.3390/jcm10163642 (PMC8396866; doi:10.3390/jcm10163642)
Supplement: Supplementary file 1 [file jcm-10-03642-s001.zip › COREAAMI_1YRSurvivor_SupplTable_Final.pdf]

Supplementary Table S1. Medications prescribed to patients.

Medication	Frequency
Discharge medication (n = 5936)	
Aspirin	5857 (98.7)
Clopidogrel	4681 (78.9)
Ticagrelor	542 (9.1)
Prasugrel	703 (11.8)
Potent $P2Y_{12}$ inhibitor	1244 (21.0)
Dual antiplatelet therapy	5848 (98.5)
Statin	5590 (94.2)
Beta-blocker	5128 (86.4)
Renin-angiotensin system inhibitor	4467 (75.3)
Medication at 1 year (n = 5397)	
Dual antiplatelet therapy	3379 (66.5)
Statin	4571 (84.7)
Beta-blocker	3846 (71.3)
Renin-angiotensin system inhibitor	3513 (65.1)

Data are shown as number (percent)

Supplementary Table S2. Clinical outcomes according to patient age and time periods after percutaneous coronary intervention

Outcomes	0–1 year	1–7 years	1–2 years	2–3 years	3–4 years	4–5 years	5–6 years	6–7 years
**Age <55 years**								
Major adverse cardiovascular event*	112/1640 (7.0)	170/1450 (18.3)	56/1450 (4.0)	29/1347 (2.2)	24/1268 (2.2)	28/977 (3.4)	17/643 (3.4)	10/375 (3.8)
All-cause death	31/1640 (1.9)	42/1530 (4.4)	11/1530 (0.7)	9/1471 (0.6)	9/1410 (0.8)	6/1107 (0.6)	5/753 (0.8)	2/447 (0.9)
Cardiovascular death	29/1640 (1.8)	36/1530 (3.5)	10/1530 (0.7)	8/1471 (0.6)	8/1410 (0.6)	5/1107 (0.5)	4/753 (0.6)	1/447 (0.5)
Non-fatal myocardial infarction	23/1640 (1.5)	41/1509 (4.5)	11/1509 (0.8)	4/1441 (0.3)	5/1379 (0.4)	10/1079 (1.1)	10/729 (1.8)	1/429 (0.3)
Non-fatal ischemic stroke	9/1640 (0.6)	12/1522 (1.5)	4/1522 (0.2)	0/1459 (0)	3/1398 (0.3)	2/1096 (0.2)	0/742 (0)	3/440 (0.9)
Any revascularization	70/1640 (4.5)	121/1463 (12.6)	47/1463 (3.3)	20/1363 (1.5)	13/1286 (1.1)	23/994 (2.8)	12/654 (2.4)	6/383 (2.2)
Stent thrombosis†	13/1640 (0.8)	9/1522 (1.1)	1/1522 (0.1)	2/1463 (0.1)	1/1401 (0.1)	3/1100 (0.3)	1/749 (0.2)	1/446 (0.3)
BARC 2, 3, and 5 bleeding	61/1640 (3.8)	45/1475 (4.5)	13/1475 (0.9)	5/1405 (0.3)	12/1345 (1.0)	10/1057 (1.1)	3/723 (0.5)	2/429 (0.8)
**Age 55–64 years**								
Major adverse cardiovascular event*	175/1514 (11.8)	205/1268 (24.3)	69/1268 (5.6)	39/1174 (3.4)	38/1092 (4)	33/787 (4.8)	14/555 (3.3)	12/320 (6.0)
All-cause death	80/1514 (5.3)	90/1367 (11.6)	23/1367 (1.7)	18/1319 (1.3)	16/1260 (1.5)	12/934 (1.5)	11/668 (2.3)	10/383 (3.8)
Cardiovascular death	72/1514 (4.8)	69/1367 (9.0)	20/1367 (1.5)	14/1319 (1.0)	12/1260 (1.2)	8/934 (1.0)	7/668 (1.5)	8/383 (3.1)
Non-fatal myocardial infarction	19/1514 (1.3)	33/1350 (3.6)	10/1350 (0.8)	8/1294 (0.6)	6/1229 (0.5)	6/904 (0.7)	1/645 (0.2)	2/370 (0.9)
Non-fatal ischemic stroke	17/1514 (1.2)	19/1350 (2.3)	4/1350 (0.3)	2/1300 (0.1)	5/1239 (0.5)	3/916 (0.3)	5/657 (1.0)	0/377 (0)
Any revascularization	81/1514 (5.7)	133/1291 (15.0)	49/1291 (3.9)	24/1199 (2.0)	27/1120 (2.8)	22/809 (3.2)	4/571 (0.9)	7/327 (3.4)
Stent thrombosis†	11/1514 (0.7)	6/1359 (0.6)	2/1359 (0.1)	0/1310 (0)	2/1251 (0.2)	2/925 (0.2)	0/663 (0)	0/379 (0)
BARC 2, 3, and 5 bleeding	81/1514 (5.6)	55/1297 (5.7)	26/1297 (2.0)	11/1231 (0.9)	9/1165 (0.8)	6/873 (0.8)	1/628 (0.3)	2/363 (1.0)
**Age 65–74 years**								
Major adverse cardiovascular event*	249/1688 (15.1)	257/1354 (27.9)	93/1354 (7.0)	47/1205 (4.0)	49/1091 (5.1)	37/798 (5.4)	23/523 (5.5)	8/265 (4.8)
All-cause death	182/1688 (10.9)	223/1448 (22.9)	59/1448 (4.1)	47/1355 (3.5)	40/1251 (3.6)	45/941 (5.7)	24/626 (4.7)	8/322 (3.7)
Cardiovascular death	148/1688 (8.9)	150/1448 (16.5)	35/1448 (2.5)	34/1355 (2.5)	28/1251 (2.6)	29/941 (3.6)	18/626 (3.6)	6/322 (2.9)
Non-fatal myocardial infarction	29/1688 (1.9)	33/1421 (3.9)	14/1421 (1.0)	3/1316 (0.2)	8/1210 (0.8)	3/903 (0.3)	4/604 (0.9)	1/311 (0.6)

Non-fatal ischemic stroke	15/1688 (1.0)	24/1436 (2.3)	5/1436 (0.3)	6/1339 (0.5)	8/1231 (0.7)	4/922 (0.5)	1/609 (0.2)	0/309 (0)
Any revascularization	83/1688 (5.5)	117/1374 (12.3)	60/1374 (4.5)	15/1230 (1.3)	18/1117 (1.8)	13/820 (1.8)	8/537 (1.8)	3/276 (1.7)
Stent thrombosis†	20/1688 (1.2)	5/1435 (0.6)	0/1435 (0)	0/1342 (0)	2/1239 (0.2)	3/930 (0.4)	0/618 (0)	0/318 (0)
BARC 2, 3, and 5 bleeding	131/1688 (8.2)	55/1349 (6.8)	19/1349 (1.4)	12/1247 (1.0)	6/1147 (0.6)	9/863 (1.2)	7/580 (1.9)	2/296 (0.8)
**Age ≥75 years**								
Major adverse cardiovascular event*	326/1400 (23.9)	302/989 (55.5)	83/989 (8.6)	62/854 (7.5)	70/729 (11.3)	42/474 (11.1)	24/275 (11.8)	21/132 (24.3)
All-cause death	300/1400 (21.7)	329/1052 (54.2)	88/1052 (8.5)	72/937 (7.8)	75/815 (10.5)	38/548 (8.7)	30/329 (11.9)	26/160 (24.6)
Cardiovascular death	258/1400 (18.9)	239/1052 (45.2)	58/1052 (5.7)	51/937 (5.7)	58/815 (8.4)	27/548 (6.2)	24/329 (9.8)	21/160 (20.6)
Non-fatal myocardial infarction	21/1400 (1.7)	23/1037 (4.0)	5/1037 (0.5)	6/920 (0.7)	2/796 (0.3)	9/534 (2.1)	1/317 (0.4)	0/154 (0)
Non-fatal ischemic stroke	25/1400 (2.0)	25/1031 (4.4)	7/1031 (0.8)	5/915 (0.6)	5/792 (0.7)	6/531 (1.4)	2/316 (1.0)	0/151 (0)
Any revascularization	46/1400 (4.0)	62/1012 (10.6)	23/1012 (2.3)	11/881 (1.3)	12/757 (2.0)	14/495 (3.7)	1/289 (0.4)	1/141 (1.3)
Stent thrombosis†	14/1400 (1.1)	9/1047 (1.3)	3/1047 (0.3)	3/932 (0.3)	1/809 (0.2)	2/543 (0.5)	0/323 (0)	0/156 (0)
BARC 2, 3, and 5 bleeding	133/1400 (10.5)	70/964 (12.1)	28/964 (3.0)	15/843 (1.8)	15/727 (2.4)	4/491 (1.0)	7/292 (3.1)	1/143 (1.2)

Data are shown as event number/number at risk (Kaplan-Meier estimates)

Abbreviation: BARC, Bleeding Academic Research Consortium

*A composite of cardiovascular death, non-fatal myocardial infarction, non-fatal ischemic stroke, and any revascularization

†Definite or probable stent thrombosis

Supplementary Table S3. Univariate Cox regression analysis of factors associated with major adverse cardiovascular events and all-cause death more than 1 year after percutaneous coronary intervention

Variables	**Major adverse cardiovascular event**		Variables	**All-cause death**	
	Unadjusted HR (95% CI)	*P* value		Unadjusted HR (95% CI)	*P* value
Age, years			Age, years		
<55	1		<55	1	
55–64	1.48 (1.20–1.81)	< 0.001	55–64	2.45 (1.70–3.54)	< 0.001
65–74	1.86 (1.53–2.26)	< 0.001	65–74	6.18 (4.44–8.59)	< 0.001
≥75	3.40 (2.81–4.11)	< 0.001	≥75	14.8 (10.7–20.4)	< 0.001
Male sex	0.74 (0.64–0.85)	< 0.001	Male sex	0.65 (0.55–0.76)	< 0.001
Body mass index ≥25 kg/m^2	0.80 (0.07–0.92)	0.001	Body mass index ≥25 kg/m^2	0.59 (0.50–0.70)	< 0.001
Hypertension	1.65 (1.44–1.88)	< 0.001	Hypertension	1.90 (1.62–2.22)	< 0.001
Diabetes mellitus	1.62 (1.42–1.85)	< 0.001	Diabetes mellitus	1.98 (1.70–2.30)	< 0.001
Dyslipidemia	0.93 (0.79–1.10)	0.399	Dyslipidemia	0.66 (0.53–0.82)	< 0.001
Current smoker	0.73 (0.64–0.83)	< 0.001	Current smoker	0.48 (0.40–0.57)	< 0.001
Family history of coronary artery disease	0.74 (0.48–1.14)	0.171	Family history of coronary artery disease	0.32 (0.15–0.67)	0.002
Previous myocardial infarction	1.86 (1.40–2.47)	< 0.001	Previous myocardial infarction	2.07 (1.51–2.85)	< 0.001
Previous percutaneous coronary intervention	1.72 (1.37–2.15)	< 0.001	Previous percutaneous coronary intervention	1.91 (1.49–2.45)	< 0.001
Previous coronary artery bypass graft	3.14 (1.57–6.30)	0.001	Previous coronary artery bypass graft	3.85 (1.83–8.10)	< 0.001
Previous cerebrovascular accident	1.60 (1.29–1.99)	< 0.001	Previous cerebrovascular accident	2.43 (1.96–3.02)	< 0.001
Atrial fibrillation	1.76 (1.34–2.31)	< 0.001	Atrial fibrillation	2.23 (1.67–2.98)	< 0.001
Clinical presentation (NSTEMI)	1.23 (1.08–1.40)	0.002	Clinical presentation (NSTEMI)	1.42 (1.22–1.65)	< 0.001
Killip classification III–IV	1.75 (1.48–2.08)	< 0.001	Killip classification III ~ IV	2.36 (1.97–2.82)	< 0.001

Left ventricular ejection fraction <40%	2.11 (1.77–2.52)	< 0.001	Left ventricular ejection fraction <40%	3.05 (2.54–3.67)	< 0.001
Anemia	1.83 (1.60–2.09)	< 0.001	Anemia	2.67 (2.30–3.11)	< 0.001
Renal insufficiency	2.57 (2.25–2.93)	< 0.001	Renal insufficiency	4.58 (3.94–5.32)	< 0.001
Multivessel coronary artery disease	1.67 (1.46–1.90)	< 0.001	Multivessel coronary artery disease	1.55 (1.33–1.81)	< 0.001
Left anterior descending artery or left main culprit vessel	0.96 (0.85–1.09)	0.556	Left anterior descending artery or left main culprit vessel	1.10 (0.94–1.27)	0.229
Number of coronary arteries treated ≥2	1.09 (0.95–1.26)	0.199	Number of coronary arteries treated ≥2	1.07 (0.91–1.25)	0.43
Total stent number ≥3	1.07 (0.89–1.28)	0.46	Total stent number ≥3	1.02 (0.82–1.25)	0.884
Mean stent diameter <3 mm	1.42 (1.24–1.62)	< 0.001	Mean stent diameter <3 mm	1.46 (1.25–1.71)	< 0.001
Total stent length >60 mm	1.07 (0.87–1.32)	0.494	Total stent length >60 mm	1.01 (0.79–1.29)	0.931
Uncontrolled systolic blood pressure	1.75 (1.48–2.07)	< 0.001	Uncontrolled systolic blood pressure	2.83 (2.36–3.38)	< 0.001
Uncontrolled LDL-cholesterol	1.39 (1.18–1.64)	< 0.001	Uncontrolled LDL-cholesterol	1.23 (0.97–1.55)	0.081

Supplementary Table S4. **A**djusted hazard ratios of factors associated with major adverse cardiovascular events and all-cause death more than 1 year after percutaneous coronary intervention according to patient age

Variables	Age <65 years		Variables	Age ≥65 years	
	Adjusted* HR (95% CI)	P value		Adjusted* HR (95% CI)	P value
**Major adverse cardiovascular events**			**Major adverse cardiovascular events**		
Renal insufficiency	2.10 (1.49–2.95)	< 0.001	Uncontrolled systolic blood pressure	1.57 (1.19–2.07)	0.001
Multivessel coronary artery disease	1.90 (1.41–2.56)	< 0.001	Age ≥75 year old†	1.46 (1.16–1.84)	0.001
Anemia	1.50 (1.12–2.00)	0.006	Left ventricular ejection fraction <40%	1.55 (1.15–2.09)	0.004
Uncontrolled LDL-cholesterol	1.39 (1.08–1.79)	0.010	Uncontrolled LDL-cholesterol	1.37 (1.09–1.73)	0.008
			Killip classification III ~ IV	1.36 (1.03–1.80)	0.030
			Multivessel coronary artery disease	1.38 (1.05–1.81)	0.021
**All-cause death**			**All-cause death**		
Uncontrolled systolic blood pressure	3.31 (2.08–5.28)	< 0.001	Uncontrolled systolic blood pressure	2.05 (1.52–2.77)	< 0.001
Left ventricular ejection fraction <40%	3.18 (1.99–5.08)	< 0.001	Age ≥75 year old†	1.91 (1.46–2.49)	<0.001
Diabetes mellitus	2.13 (1.41–3.20)	< 0.001	Renal insufficiency	1.84 (1.4–2.43)	< 0.001
Anemia	1.83 (1.19–2.82)	0.006	Male sex	1.82 (1.38–2.42)	< 0.001
Body mass index ≥25 kg/m^2	0.59 (0.38–0.89)	0.013	Left ventricular ejection fraction <40%	1.82 (1.30–2.54)	< 0.001
			Killip classification III–IV	1.53 (1.12–2.1)	0.008
			Uncontrolled LDL-cholesterol	1.33 (1.01–1.76)	0.044

Abbreviations: CI, confidence interval; HR, hazard ratio; LDL, low-density lipoprotein; NSTEMI, non-ST-segment elevation myocardial infarction.

*Adjusted by all covariates in Table 1 with *P* values <0.1 on univariate analysis

†Compared to age 65~74 year old
